# Complete genome of *Phenylobacterium zucineum *– a novel facultative intracellular bacterium isolated from human erythroleukemia cell line K562

**DOI:** 10.1186/1471-2164-9-386

**Published:** 2008-08-13

**Authors:** Yingfeng Luo, Xiaoli Xu, Zonghui Ding, Zhen Liu, Bing Zhang, Zhiyu Yan, Jie Sun, Songnian Hu, Xun Hu

**Affiliations:** 1 Cancer Institute (Key Laboratory for Cancer Intervention and Prevention, National Ministry of Education, PR China; Key Laboratory of Molecular Biology in Medical Sciences, Zhejiang Province, PR China), the Second Affiliated Hospital, Zhejiang University School of Medicine, Hangzhou, PR China; 2 James D. Watson Institute of Genome Sciences, Zhejiang University, Hangzhou, PR China; 3 Key Laboratory of Genome Sciences and Information, Beijing Institute of Genomics, Chinese Academy of Sciences, Beijing, PR China

## Abstract

**Background:**

*Phenylobacterium zucineum *is a recently identified facultative intracellular species isolated from the human leukemia cell line K562. Unlike the known intracellular pathogens, *P. zucineum *maintains a stable association with its host cell without affecting the growth and morphology of the latter.

**Results:**

Here, we report the whole genome sequence of the type strain HLK1^T^. The genome consists of a circular chromosome (3,996,255 bp) and a circular plasmid (382,976 bp). It encodes 3,861 putative proteins, 42 tRNAs, and a 16S-23S-5S rRNA operon. Comparative genomic analysis revealed that it is phylogenetically closest to *Caulobacter crescentus*, a model species for cell cycle research. Notably, *P. zucineum *has a gene that is strikingly similar, both structurally and functionally, to the cell cycle master regulator CtrA of *C. crescentus*, and most of the genes directly regulated by CtrA in the latter have orthologs in the former.

**Conclusion:**

This work presents the first complete bacterial genome in the genus *Phenylobacterium*. Comparative genomic analysis indicated that the CtrA regulon is well conserved between *C. crescentus *and *P. zucineum*.

## Background

*Phenylobacterium zucineum *strain HLK1^T ^is a facultative intracellular microbe recently identified by us [1]. It is a rod-shaped Gram-negative bacterium 0.3–0.5 × 0.5–2 μm in size. It belongs to the genus *Phenylobacterium *[2], which presently comprises 5 species, *P. lituiforme *(FaiI3T) [3], *P. falsum *(AC49T) [4], *P. immobile *(ET) [2], *P. koreense *(Slu-01T) [5], and *P. zucineum *(HLK1^T^) [1]. They were isolated from subsurface aquifer, alkaline groundwater, soil, activated sludge from a wastewater treatment plant, and the human leukemia cell line K562, respectively. Except for *P. zucineum*, they are environmental bacteria, and there is no evidence that these microbes are associated with eukaryotic cells. The HLK1^T ^strain, therefore, represents the only species so far in the genus *Phenylobacterium *that can infect and survive in human cells. Since most, if not all, of the known microbes that can invade human cells are pathogenic, we proposed that HLK1^T ^may have pathogenic relevance to humans [1]. Unlike the known intracellular pathogens that undergo a cycle involving invasion, overgrowth, and disruption of the host cells, and repeating the cycle by invading new cells, HLK1^T ^is able to establish a stable parasitic association with its host, i.e., the strain does not overgrow intracellularly to kill the host, and the host cells carry them to their progeny. One cell line (SW480) infected with *P. zucineum *has been stably maintained for nearly three years in our lab (data not shown).

In this report, we present the complete genome sequence of *P. zucineum*.

## Results

### Genome anatomy

**Figure 1:**
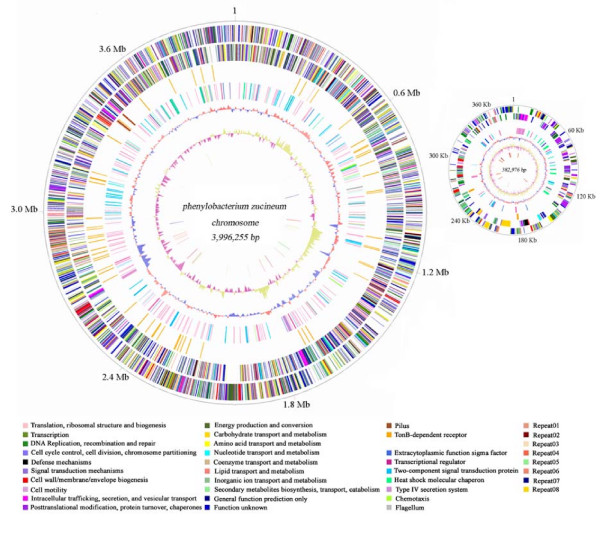
**Circular representation of the *P. zucineum *strain HLK1^T ^chromosome and plasmid (smaller circle)**. Circles indicate (from the outside): (1) Physical map scaled in megabases from base 1, the start of the putative replication origin. (2) Coding sequences transcribed in the clockwise direction are color-coded according to COG functional category. (3) Coding sequences transcribed in the counterclockwise direction are color-coded according to COG functional category. (4) Proteins involved in establishment of intracellular niche are TonB-dependent receptors (orange) and pilus genes (sienna). (5) Functional elements responsible for environmental transition are extracytoplasmic function sigma factors (royal blue), transcriptional regulators (violet red), two-component signal transduction proteins (deep sky blue), heat shock molecular chaperons (spring green), type IV secretion systems (plum), chemotaxis systems (green yellow) and flagellum proteins (gray). (6) G + C percent content (10-kb window and 1-kb incremental shift for chromosome; 300 bp window and 150 bp for incremental shift for plasmid); values larger than average (71.35% in chromosome and 68.5% in plasmid) are in red and smaller in medium blue. (7) GC skew (10-kb window and 1-kb incremental shift for chromosome; 300 bp window and 150 bp for incremental shift for plasmid); values greater than zero are in gold and smaller in purple. (8) Repeat families, repeats 01-08 are in dark salmon, dark red, wheat, tomato, light green, salmon, dark blue and gold, respectively.

The genome is composed of a circular chromosome (3,996,255 bp) and a circular plasmid (382,976 bp) (Figure 1; Table 1). The G + C contents of chromosome and plasmid are 71.35% and 68.5%, respectively. There are 3,861 putative protein-coding genes (3,534 in the chromosome and 327 in the plasmid), of which 3,180 have significant matches in the non-redundant protein database. Of the matches, 585 are conserved hypothetical proteins and 2,595 are proteins with known or predicted functions. Forty-two tRNA genes and one 16S-23S-5S rRNA operon were identified in the chromosome.

**Table 1:** Genome summary of *P. zucineum *Strain *HLK1*^T^

Genomic Element		Chromosome	plasmid
Length (bp)		3,996,255	382,976
GC content (%)		71.35	68.54
Proteins		3, 534	327
	Coding region of genome (%)	88.85%	81.94%
	Proteins with known or predicted function	2,394(67.75%)	201(61.47%)
	Conserved hypothetical proteins	560(15.84%)	25(7.65%)
	Hypothetical proteins	580(16.41%)	101(30.88%)
rRNA operon		1	0
tRNAs		42	0
			
Proteins in each	[J] Translation, ribosomal structure and biogenesis	185 (5.24%)	3 (1.21%)
COG category	[K] Transcription	210 (5.94%)	22 (8.91%)
	[L] Replication, recombination and repair	139 (3.93%)	23 (9.31%)
	[D] Cell cycle control, cell division, chromosome partitioning	27 (0.76%)	0
	[V] Defense mechanisms	51 (1.44%)	3 (1.21%)
	[T] Signal transduction mechanisms	166 (4.7%)	24 (9.72%)
	[M] Cell wall/membrane/envelope biogenesis	195 (5.52%)	15 (6.07%)
	[N] Cell motility	62 (1.75%)	4 (1.62%)
	[U] Intracellular trafficking, secretion, and vesicular transport	96 (2.72%)	13 (5.26%)
	[O] Posttranslational modification, protein turnover, chaperones	151 (4.27%)	32 (12.96%)
	[C] Energy production and conversion	188 (5.32%)	16 (6.48%)
	[G] Carbohydrate transport and metabolism	161 (4.56%)	15 (6.07%)
	[E] Amino acid transport and metabolism	293 (8.29%)	5 (2.02%)
	[F] Nucleotide transport and metabolism	58 (1.64%)	3 (1.21%)
	[H] Coenzyme transport and metabolism	116 (3.28%)	3 (1.21%)
	[I] Lipid transport and metabolism	215 (6.09%)	12 (4.86%)
	[P] Inorganic ion transport and metabolism	223 (6.31%)	24 (9.72%)
	[Q] Secondary metabolites biosynthesis, transport and catabolism	152(4.3%)	9 (3.64%)
	[R] General function prediction only	444 (12.57%)	28 (11.34%)
	[S] Function unknown	307 (8.69%)	20 (8.10%)

There are 7 families of protein-coding repetitive sequences and a family of noncoding repeats in the genome (Table 2). Notably, identical copies of repeats 02–04 were found in both the chromosome and the plasmid, suggesting their potential involvement in homologous recombination.

**Table 2:** Repetitive elements in the *P. zucineum *genome

Repeat ID	Length bp	DR^1^	Number of copies		Position of insertion		Identity (%)	Coding information
					
			Complete^2^	Partial	Chromosome	Plasmid		
Repeat01^3^	2,587	7	3	1	0	4	>99	Transposase
Repeat02^4^	1,262	3	3	1	2	2	100	Transposase
Repeat03^5^	1,392	NA	4	2	4	2	100	Transposase
Repeat04^6^	1,257	NA	10	0	7	3	100	Transposase
Repeat05	1,554	NA	2	0	2	0	>98	Hypothetical protein
Repeat06	1,136	NA	2	0	2	0	>90	Isovaleryl-CoA dehydrogenase
Repeat07	1,077	NA	2	0	2	0	>98	2-nitropropane dioxygenase
Repeat08	≈130	NA	13	0	13	0	>90	Noncoding repeats

^1^Size in base pairs of the consensus and the direct repeat (DR) generated by insertion into the genome target site.

^2^A copy is complete if the length of the repeat is ≥ 90% of the consensus, otherwise, the copy is partial.

^3^One complete copy which harbors a 7 bp direct repeat (TCCTAAC) that disrupts the VirD4.

^4^The partial copy is located in the plasmid.

^5^Two partial copies are located in the chromosome, of which a "partial" copy with full length is inserted by a copy of repeat04.

^6^repeats 01–04 are IS elements

On the basis of COG (Cluster of Orthologous Groups) classification, the chromosome is enriched in genes for basic metabolism, such as categories E (amino acid transport and metabolism) and I (lipid transport and metabolism), accounting for 8.29% and 6.09% of the total genes in the chromosome, respectively. On the other hand, the plasmid is enriched for genes in categories O (posttranslational modification, protein turnover, chaperones) and T (signal transduction mechanisms), constituting 12.96% and 9.72% of the total genes in the plasmid, respectively.

As to genes in the plasmid that cope with environmental stimuli, about half of the genes in category O are molecular chaperones (17/32), including 2 *dnaJ*-like molecular chaperones, 2 clusters of *dnaK *and its co-chaperonin *grpE *(PHZ_p0053-0054 and PHZ_p0121-122), a cluster of *groEL *and its co-chaperonin *groES *(PHZ_p0095-0096), and 9 heat shock proteins Hsp20. Of 23 genes in category T, there is one cluster (FixLJ, PHZ_p0187-0188), which is essential for the growth of *C. crescentus *under hypoxic conditions [6].

### General metabolism

The enzyme sets of glycolysis and the Entner-Doudoroff pathway are complete in the genome. All genes comprising the pentose phosphate pathway except gluconate kinase were identified, consistent with our previous experimental result that the strain cannot utilize gluconate [1]. The genome lacks two enzymes (*kdh*, alpha ketoglutarate dehydrogenase and *kgd*, alpha ketoglutarate decarboxylase), making the oxidative and reductive branches of the tricarboxylic acid cycle operate separately. The genome has all the genes for the synthesis of fatty acids, 20 amino acids, and corresponding tRNAs. Although full sets of genes for the biosynthesis of purine and pyrimidine were identified, enzymes for the salvage pathways of purine (*apt*, adenine phosphoribosyltransferase; *ade*, adenine deaminase) and pyrimidine (*cdd*, cytidine deaminase; *codA*, cytosine deaminase; *tdk*, thymidine kinase; *deoA*, thymidine phosphorylase; *upp*, uracil phosphoribosyltransferase; *udk*, uridine kinase; and *udp*, uridine phosphorylase) were absent. The plasmid encodes some metabolic enzymes, such as those participating in glycolysis, the pentose phosphate pathway, and the citric acid cycle. However, it is worth noting that the plasmid has a gene (6-phosphogluconate dehydrogenase) that is the only copy in the genome (PHZ_p0183).

Like most other species in the genus *Phenylobacterium*, the strain is able to use L-phenylalanine as a sole carbon source under aerobic conditions [1]. A recent study revealed that phenylalanine can be completely degraded through the homogentisate pathway in *Pseudomonas putida *U [7]. *P. zucineum *may use the same strategy to utilize phenylalanine, because all the enzymes for the conversion of phenylalanine through intermediate homogentisate to the final products fumarate and acetoacetate are present in the chromosome (Table 3).

**Table 3:** Phenylalanine-degrading enzymes in the *P. zucineum *genome

Gene	*P. zucineum *Locus	Length (bp)		Alignment coverage (%)		Score	Amino acid Identity (%)	Gene name
					
		*P. putida*	*P. zucineum*	*P. putida*	*P. zucineum*			
*phhA*	PHZ_c1409	262	308	83.59	71.75	219	48.65	phenylalanine-4-hydroxylase
*phhB*	PHZ_c0077	118	97	79.66	93.81	38.5	26.32	carbinolamine dehydratase
*tryB*	PHZ_c1644	398	406	60.05	57.39	33.9	21.86	tyrosine aminotransferase
*hpd*	PHZ_c2833	358	374	98.32	93.58	398	57.98	4-hydroxyphenylpyruvate dioxygenase
*hmgA*	PHZ_c2831	433	377	60.28	67.64	53.5	22.3	homogentisate 1,2-dioxygenase
*hmgB*	PHZ_c0313	430	226	9.77	18.14	27.7	39.53	fumarylacetoacetate hydrolase
*hmgC*	PHZ_c0314	210	212	98.1	98.11	213	51.67	maleylacetoacetate isomerase

### Functional elements responding to environmental transition

**Figure 2:**
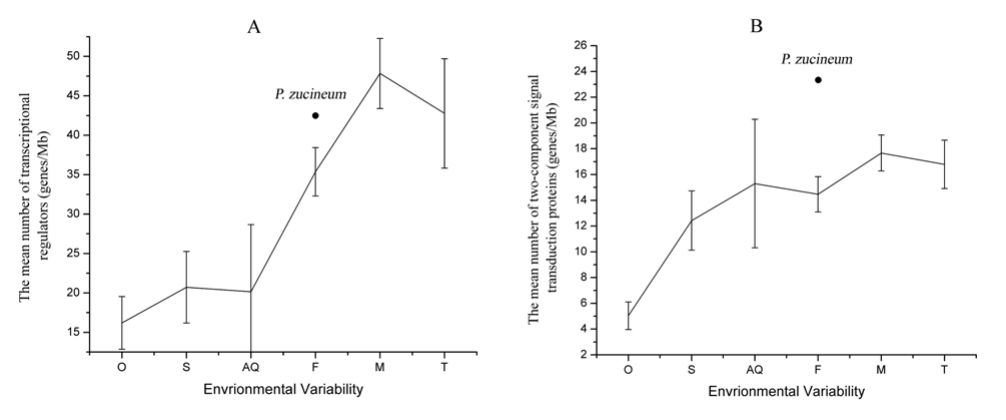
**Comparative analysis of transcriptional regulators and two-component signal transduction proteins in 6 groups of bacteria classified according to their habitats**. (A): The mean number of transcriptional regulators in each megabase pair of the genomes. (B): The mean number of two-component signal transduction proteins in each megabase pair of the genomes. The fraction of transcriptional regulators and two-component signal transduction proteins (solid black circle) of *P. zucineum *were 41.56 genes/Mb and 23.30 genes/Mb, respectively. Error bars represent standard errors. O: Obligate (26 species), S: Specialized (5 species), AQ: Aquatic (4 species), F: Facultative (28 species), M: Multiple (27 species), T: Terrestrial (3 species).

HLK1^T ^is able to survive intracellularly and extracellularly. Consistently, the genome contains the fundamental elements to support the life cycle in different environments. The genome contains abundant two-component signal transduction proteins, transcriptional regulators, and heat shock response proteins, enabling the strain to respond to extra- and intra-cellular stimuli at transcriptional and post-translational levels. Among the total of 102 two-component signal transduction proteins (91 in the chromosome and 11 in the plasmid), there are 36 histidine kinases, 48 response regulators, and 18 hybrid proteins fused with histidine kinase and response regulator. Sixteen pairs of histidine kinase and response regulator (1 in the plasmid) are adjacently aligned and may act as functional operons. These tightly linked modules make two-component signal transduction systems respond to environmental changes efficiently. The genome encodes 170 transcriptional regulators (16 in the plasmid) (Table 4). Notably, we annotated the proteins of 93 bacteria (see methods – comparative genomics) with the same annotation criteria used for *P. zucineum *and found that the fraction of two-component signal transduction proteins and transcriptional regulators was positively correlated with the capacity for environmental adaptation (Figure 2). The genome contains 17 extracytoplasmic function (ECF) sigma factors (3 in the plasmid) (Table 5). ECFs are suggested to play a role in environmental adaptation for *Pseudomonas putida *KT2440, whose genome contains 19 ECFs [8]. *P. zucineum *has 3 heat shock sigma factors *rpoH *(2 in the plasmid) and 33 heat shock molecular chaperons (17 in the plasmid) (Table 6), which can cope with a variety of stresses, including cellular energy depletion, extreme concentrations of heavy metals, and various toxic substances. [9].

**Table 4:** Transcriptional regulators in the *P. zucineum *genome

Family name	Action type	Chromosome	Plasmid	Proposed roles
AsnC family	Activator/repressor	8	0	Amino acid biosynthesis
AraC family	Activator	10	1	Carbon metabolism, stress response and pathogenesis
ArsR family	Repressor	8	0	Metal resistance
BlaI family	Repressor	2	0	Penicillin resistance
Cold shock family	Activator	6	0	Low-temperature resistance
Cro/CI family	Repressor	9	2	Unknown^2^
Crp/Fnr family	Activator/repressor	7	2	Global responses, catabolite repression and anaerobiosis
GntR family	Repressor	7	0	General metabolism
LacI family	Repressor	4	0	Carbon source utilization
LuxR family	Activator	5	1	Quorum sensing, biosynthesis and metabolism, etc.
LysR family	Activator/repressor	15	1	Carbon and nitrogen metabolism
MarR family	Activator/repressor	6	0	Multiple antibiotic resistance
MerR family	Repressor	9	2	Resistance and detoxification
TetR family	Repressor	22	0	Biosynthesis of antibiotics, efflux pumps, osmotic stress, etc.
XRE family	Repressor	2	2	Unknown (initial function is lysogeny maintenance)
Other types^2^	-	34	5	-
Total	-	154	16	-

^1^Initial function is related to controlling the expression of phage gene

^2^"Other types" include the transcriptional regulators with only one member in the *P. zucineum *genome or transcriptional regulators that could not be classified into any known family.

**Table 5:** Extracytoplasmic function (ECF) sigma factors in the *P. zucineum *genome

Locus	Location of proteins			COG category
		
	Genomic element	5'-end	3'-end	
PHZ_p0151	Plasmid	171,032	170,316	COG1595^1^
PHZ_p0174	Plasmid	208,703	208,053	COG1595
PHZ_p0192	Plasmid	229,133	228,516	COG1595
PHZ_c0249	Chromosome	249,840	250,553	COG1595
PHZ_c0301	Chromosome	296,299	295,706	COG1595
PHZ_c1475	Chromosome	1,676,920	1,677,492	COG1595
PHZ_c1529	Chromosome	1,730,783	1,731,403	COG1595
PHZ_c1531	Chromosome	1,732,219	1,732,800	COG1595
PHZ_c1907	Chromosome	2,134,971	2,135,507	COG1595
PHZ_c2171	Chromosome	2,447,581	2,448,396	COG1595
PHZ_c2233	Chromosome	2,526,836	2,527,369	COG1595
PHZ_c2394	Chromosome	2,724,759	2,725,307	COG1595
PHZ_c2577	Chromosome	2,965,250	2,964,390	COG1595
PHZ_c2585	Chromosome	2,970,368	2,969,811	COG1595
PHZ_c2684	Chromosome	3,077,272	3,076,727	COG1595
PHZ_c0569	Chromosome	605,441	604,233	COG4941^2^
PHZ_c3154	Chromosome	3,582,010	3,583,269	COG4941

^1^COG1595, DNA-directed RNA polymerase specialized sigma subunit, sigma24 homolog;

^2^COG4941, predicted RNA polymerase sigma factor containing a TPR repeat domain

**Table 6:** Distribution of heat shock related proteins in *P. zucineum *and representative alphaproteobacteria with different living habitats

Content\Species	*S. meliloti*	*B. suis*	*C. crescentus*	*P. zucineum*		*R. conorii*	*G. oxydans*
						
				Chromosome	Plasmid		
*rpoH*, heat shock sigma factor^1^	2	2	1	1	2	1	1
*dnaK*, molecular chaperone^2 ^(Hsp70)	1	1	1	1	2	1	1
*grpE*, molecular chaperone (co-chaperonin of Hsp70)	1	1	1	1	2	1	1
dnaK-like molecular chaperone	1	1	1	1	0	1	1
dnaJ, molecular chaperone	1	1	1	1	0	1	1
dnaJ-like molecular chaperone	4	3	3	6	2	1	3
*groEL*, molecular chaperone (hsp60)	5	1	1	1	1	1	1
*groES*, molecular chaperone (Hsp10, co-chaperonin of Hsp60)	3	1	1	1	1	1	1
molecular chaperone Hsp20	5	2	2	3	9	0	3
molecular chaperone Hsp33	1	1	1	1	0	0	1

^1^rpoH may be responsible for the expression of some or all heat shock proteins

^2^The function of molecular chaperones is to protect unfolded proteins induced by stress factors through renaturation or degradation in cooperation with protease.

The genes for cell motility include 3 chemotaxis operons, 7 MCP (methyl-accepting chemotaxis) genes, 15 other genes related to chemotaxis (Table 7), and 43 genes for the biogenesis of the flagellum (Table 8).

**Table 7:** Chemotaxis proteins in the *P. zucineum *genome

Locus *P. zucineum*	5'-end	3'-end	Name	Orthologs *C. crescentus*	Operon	Best BLAST match
PHZ_c0690	753,270	753,812	chemotaxis protein CheW	-	1	*M. magneticum AMB-1*
PHZ_c0691	753,812	755,218	chemotaxis protein methyltransferase CheR	-	1	*M. magnetotacticum MS-1*
PHZ_c0692	755,240	755,836	chemotaxis signal transduction protein	-	1	*Rhodospirillum centenum*
PHZ_c0693	755,836	757,488	methyl-accepting chemotaxis protein	-	1	*M. magneticum AMB-1*
PHZ_c0694	757,501	759,642	chemotaxis histidine kinase CheA	-	1	*M. magnetotacticum MS-1*
PHZ_c0695	759,642	760,709	chemotaxis response regulator CheB	-	1	*Rhodospirillum centenum*
PHZ_c3230	3,661,514	3,661,050	CheE protein	-	2	*C. crescentus CB15*
PHZ_c3231	3,662,099	3,661,527	chemotaxis protein CheYIII	CC0440	2	*C. crescentus CB15*
PHZ_c3233	3,662,860	3,662,477	chemotaxis protein CheYII	CC0591	2	*R. palustris CGA009*
PHZ_c3234	3,663,186	3,666,188	chemotaxis histidine kinase CheA	CC0594	2	*Azospirillum brasilense*
PHZ_c3235	3,666,188	3,666,733	chemotaxis protein CheW	CC0595	2	*Rhodospirillum centenum*
PHZ_c3236	3,666,786	3,669,191	methyl-accepting chemotaxis protein McpH	CC3349	2	*R. palustris CGA009*
PHZ_c3237	3,670,166	3,669,336	chemotaxis protein methyltransferase CheR	CC0598	2	*R. palustris HaA2*
PHZ_c3238	3,671,242	3,670,166	chemotaxis response regulator CheB	CC0597	2	*M. magneticum AMB-1*
PHZ_c3371	3,820,121	3,819,669	CheE protein	CC0441	3	*C. crescentus CB15*
PHZ_c3372	3,820,729	3,820,124	chemotaxis protein CheYIII	-	3	*C. crescentus CB15*
PHZ_c3373	3,821,034	3,820,729	CheU protein	CC0439	3	*C. crescentus CB15*
PHZ_c3374	3,821,651	3,821,082	chemotaxis protein CheD	CC0438	3	*C. crescentus CB15*
PHZ_c3375	3,822,037	3,821,651	chemotaxis protein CheYII	CC0437	3	*C. crescentus CB15*
PHZ_c3376	3,823,068	3,822,040	chemotaxis response regulator CheB	CC0436	3	*C. crescentus CB15*
PHZ_c3377	3,823,955	3,823,068	chemotaxis protein methyltransferase CheR	CC0435	3	*A. cryptum JF-5*
PHZ_c3378	3,824,410	3,823,946	chemotaxis protein CheW	CC0434	3	*Rhizobium etli CFN 42*
PHZ_c3379	3,826,614	3,824,422	chemotaxis histidine kinase CheA	CC0433	3	*A. cryptum JF-5*
PHZ_c3380	3,826,997	3,826,635	chemotaxis protein CheYI	CC0432	3	*Caulobacter vibrioides*
PHZ_c3381	3,827,299	3,826,997	CheX protein	CC0431	3	*Sinorhizobium meliloti*
PHZ_c3382	3,829,234	3,827,306	methyl-accepting chemotaxis protein McpA	CC0430	3	*A. cryptum JF-5*
PHZ_c0101	94,220	93,750	CheE protein	-	scatted	*C. crescentus CB15*
PHZ_c0102	94,795	94,220	chemotaxis protein CheYIII	-	scatted	*C. crescentus CB15*
PHZ_c0297	292,469	292,864	chemotaxis protein CheYIV	CC3471	scatted	*C. crescentus CB15*
PHZ_c0298	292,867	293,679	chemotaxis protein methyltransferase CheR	CC3472	scatted	*C. crescentus CB15*
PHZ_c0732	803,383	804,876	methyl-accepting chemotaxis protein McpB	CC0428	scatted	*C. crescentus CB15*
PHZ_c0961	1,057,134	1,058,720	methyl-accepting chemotaxis protein McpI	CC2847	scatted	*R. palustris CGA009*
PHZ_c1198	1,380,883	1,383,294	methyl-accepting chemotaxis protein McpU	-	scatted	*A. cryptum JF-5*
PHZ_c1199	1,383,297	1,383,758	chemotaxis protein CheW1	-	scatted	*Sinorhizobium meliloti*
PHZ_c1687	1,890,274	1,891,176	chemotaxis MotB protein	CC1573	scatted	*C. crescentus CB15*
PHZ_c1936	2,169,634	2,169,939	chemotactic signal response protein CheL	CC2583	scatted	*C. crescentus CB15*
PHZ_c2211	2,499,744	2,499,274	chemotaxis protein CheYIII	-	scatted	*O. alexandrii HTCC2633*
PHZ_c2392	2,720,611	2,720,144	chemotaxis protein CheYIII	-	scatted	*C. crescentus CB15*
PHZ_c2741	3,142,750	3,143,238	chemotaxis protein CheYIII	CC3155	scatted	*C. crescentus CB15*
PHZ_c3123	3,549,150	3,550,016	chemotaxis MotA protein	CC0750	scatted	*C. crescentus CB15*
PHZ_c3401	3,848,811	3,850,766	methyl-accepting chemotaxis protein McpA	-	scatted	*C. vibrioides*

**Table 8:** Flagella genes in the *P. zucineum *genome

Locus	5'-end	3'-end	Name	Gene symbol	Proposed role
PHZ_c0080	75,413	76,462	flagellin modification protein FlmA	*flmA*	regulator
PHZ_c0081	76,467	77,621	flagellin modification protein FlmB	*flmB*	regulator
PHZ_c0745	816,772	818,034	flagellar hook-length control protein FliK	*fliK*	flagellar structure
PHZ_c0787	868,051	866,696	flagellar hook protein FlgE	*flgE*	flagellar structure
PHZ_c0788	868,860	868,171	flagellar hook assembly protein FlgD	*flgD*	flagellar structure
PHZ_c0789	870,604	868,865	flagellar hook length determination protein	*flage*	regulator
PHZ_c0790	870,819	872,918	flagellar hook-associated protein	*flaN*	flagellar structure
PHZ_c0791	872,933	873,862	flagellin and related hook-associated proteins	-	flagellar structure
PHZ_c0853	945,008	946,354	flagellum-specific ATP synthase FliI	*fliI*	protein export ATPase
PHZ_c0854	946,354	946,758	fliJ protein	*fliJ*	flagellar structure
PHZ_c0857	950,714	948,621	flagellar biosynthesis protein FlhA	*flhA*	export apparatus
PHZ_c0859	952,470	952,138	flagellar motor switch protein FliN	*fliN*	motor
PHZ_c0860	953,126	952,479	flbE protein	*flbE*	regulator
PHZ_c0861	954,151	953,126	flagellar motor switch protein FliG	*fliG*	motor
PHZ_c0862	955,794	954,151	flagellar M-ring protein FliF	*fliF*	flagellar structure
PHZ_c0913	1,007,753	1,006,992	flagellar L-ring protein FlgH	*flgH*	flagellar structure
PHZ_c0914	1,008,508	1,007,753	distal basal-body ring component protein FlaD	*flaD*	flagellar structure
PHZ_c0915	1,009,300	1,008,515	flagellar basal-body rod protein FlgG	*flgG*	flagellar structure
PHZ_c0916	1,010,052	1,009,318	flagellar basal-body rod protein FlgF	*flgF*	flagellar structure
PHZ_c0917	1,010,272	1,010,874	flagellar basal body-associated protein FliL	*fliL*	flagellar structure
PHZ_c0918	1,010,910	1,011,983	flagellar motor switch protein FliM	*fliM*	motor
PHZ_c0922	1,017,085	1,016,351	flagellar biosynthesis protein FliP	*fliP*	export apparatus
PHZ_c0923	1,017,420	1,017,151	flagellar protein FliO	*fliO*	export apparatus
PHZ_c0924	1,017,502	1,017,918	flagellar basal-body rod protein FlgB	*flgB*	flagellar structure
PHZ_c0925	1,017,942	1,018,355	flagellar basal-body rod protein FlgC	*flgC*	flagellar structure
PHZ_c0926	1,018,370	1,018,678	flagellar hook-basal body complex protein FliE	*fliE*	flagellar structure
PHZ_c0930	1,021,796	1,022,056	flagellar biosynthesis protein FliQ	*fliQ*	export apparatus
PHZ_c0931	1,022,079	1,022,837	flagellar biosynthesis protein FliR	*fliR*	export apparatus
PHZ_c0932	1,022,837	1,023,913	flagellar biosynthesis protein FlhB	*flhB*	export apparatus
PHZ_c1380	1,563,281	1,562,745	putative flagella accessory protein FlaCE	*flaCE*	flagellar structure
PHZ_c1381	1,565,145	1,563,358	flagellin modification protein FlmG	*flmG*	regulator
PHZ_c1382	1,565,343	1,565,765	flagellar repressor protein FlbT	*flbT*	regulator
PHZ_c1383	1,565,782	1,566,093	flagellar biosynthesis regulator FlaF	*flaF*	regulator
PHZ_c1384	1,566,375	1,567,202	flagellin FljM	*fljM*	flagellar structure
PHZ_c1385	1,567,469	1,568,314	flagellin FljM	*fljM*	flagellar structure
PHZ_c1386	1,568,434	1,568,724	flagellin FlaG	*flaG*	flagellar structure
PHZ_c1387	1,568,887	1,569,720	flagellin FljL	*fljL*	flagellar structure
PHZ_c1935	2,168,522	2,169,634	flagellar P-ring protein FglI	*fglI*	flagellar structure
PHZ_c1937	2,169,942	2,170,382	flagellar basal-body protein FlbY	*flbY*	flagellar structure
PHZ_c2595	2,982,550	2,983,593	flagellin modification protein FlmD	*flmD*	regulator
PHZ_c2597	2,984,874	2,986,508	flagellin modification protein FlmG	*flmG*	regulator
PHZ_c2599	2,989,315	2,989,974	flmC; flagellin modification protein FlmC	*flmC*	regulator
PHZ_c2600	2,990,549	2,989,977	flagellin modification protein FlmH	*flmH*	regulator

The genome contains sec-dependent, sec-independent, typical type II (Table 9) and IV secretion systems (Table 10), which are known to play important roles in adapting to diverse conditions [10,11].

**Table 9:** Distributions of proteins involved in environmental adaptation in *P. zucineum *and representative alphaproteobacteria with different living habitats

Species	*S. meliloti*	*B. suis*	*C. crescentus*	*P. zucineum*	*R. conorii*	*G. oxydans*
Genome size (Mb)	6.69	3.32	4.02	4.38	1.27	2.92
GC content (%)	62.2	57.3	67.2	71.1	32.4	60.8
Habitat	Multiple^1^	Facultative^1^	Aquatic^1^	Facultative^2^	Obligate^1^	Multiple^3^
ECF, extracytoplasmic function sigma factor (/Mb)	11 (1.6)	2 (0.6)	15 (3.7)	17 (3.9)	0 (0)	2 (0.7)
Transcriptional regulator (/Mb)	433 (64.7)	149(44.9)	183 (45.5)	170 (38.8)	11 (8.7)	89 (30.1)
Two-component signal transduction protein (/Mb)	113 (16.9)	44 (13.3)	111 (27.6)	102 (23.3)	7 (5.5)	41 (14.1)
molecular chaperone	23	12	14	33	8	14
Flagellar protein	41	37	42	43	10	40
Chemotaxis protein	42	4	48	41	0	11
Pilus protein	13	4	9	16	2	4
Sec-dependent secretion system	11	11	11	11	11	12
Sec-independent secretion system	4	4	4	4	3	4
Type II secretory protein	2	0	8	13	0	3
Type IV secretory protein	9	8	9	31	15	1

^1^The habitats of *S. meliloti, B. suis, and R. conorii *were indicated in a recent publication [42].

^2^According to our recent publication [1], *P. zucineum *was classified as "facultative". ^3^Given that *G. oxydans *is often isolated from sugary niches (such as flowers and fruits) and associated soil (such as garden soil and baker's soil) [43], we classified *G. oxydans *as "multiple".

**Table 10:** Type IV secretion systems in the *P. zucineum *genome

Locus	Location of protein			Name
		
	Genomic element	5'-end	3'-end	
PHZ_p0007	Plasmid	6,786	7,445	type IV secretion protein, VirB1
PHZ_p0008	Plasmid	7,483	7,800	type IV secretion protein, VirB2
PHZ_p0009	Plasmid	7,816	8,148	type IV secretion protein, VirB3
PHZ_p0010	Plasmid	8,144	10,546	type IV secretion protein, VirB4
PHZ_p0011	Plasmid	10,546	11,298	type IV secretion protein, VirB5
PHZ_p0012	Plasmid	11,553	12,488	type IV secretion protein, VirB6
PHZ_p0013	Plasmid	12,816	13,493	type IV secretion protein, VirB8
PHZ_p0014	Plasmid	13,493	14,320	type IV secretion protein, VirB9
PHZ_p0015	Plasmid	14,320	15,543	type IV secretion protein, VirB10
PHZ_p0016	Plasmid	15,543	16,538	type IV secretion protein, VirB11
PHZ_c1506	Chromosome	1,709,481	1,709,999	type IV secretion protein, TraF
PHZ_c1508	Chromosome	1,711,058	1,712,773	type IV secretion protein, VirD2
PHZ_c1509	Chromosome	1,712,790	1,714,763	type IV secretion protein, VirD4
PHZ_c1512	Chromosome	1,716,262	1,717,242	conjugal transfer protein, TrbB
PHZ_c1513	Chromosome	1,717,242	1,717,559	conjugal transfer protein, TrbC
PHZ_c1514	Chromosome	1,717,562	1,717,828	conjugal transfer protein, TrbD
PHZ_c1515	Chromosome	1,717,836	1,720,283	conjugal transfer protein, TrbE
PHZ_c1516	Chromosome	1,720,283	1,721,014	conjugal transfer protein, TrbJ
PHZ_c1517	Chromosome	1,721,238	1,722,398	conjugal transfer protein, TrbL
PHZ_c1518	Chromosome	1,722,401	1,723,084	conjugal transfer protein, TrbF
PHZ_c1519	Chromosome	1,723,087	1,724,064	conjugal transfer protein, TrbG
PHZ_c1520	Chromosome	1,724,070	1,725,212	conjugal transfer protein, TrbI
PHZ_c2348	Chromosome	2,660,517	2,660,813	type IV secretion protein, VirB2
PHZ_c2349	Chromosome	2,660,809	2,661,144	type IV secretion protein, VirB3
PHZ_c2350	Chromosome	2,661,119	2,663,497	type IV secretion protein, VirB4
PHZ_c2352	Chromosome	2,664,374	2,665,309	type IV secretion protein, VirB6
PHZ_c2353	Chromosome	2,665,482	2,666,159	type IV secretion protein, VirB8
PHZ_c2354	Chromosome	2,666,159	2,667,004	type IV secretion protein, VirB9
PHZ_c2355	Chromosome	2,667,004	2,668,041	type IV secretion protein, VirB10
PHZ_c2356	Chromosome	2,668,046	2,669,035	type IV secretion protein, VirB11
PHZ_c2357	Chromosome	2,669,091	2,670,872	type IV secretion protein, VirD4

**Figure 3:**
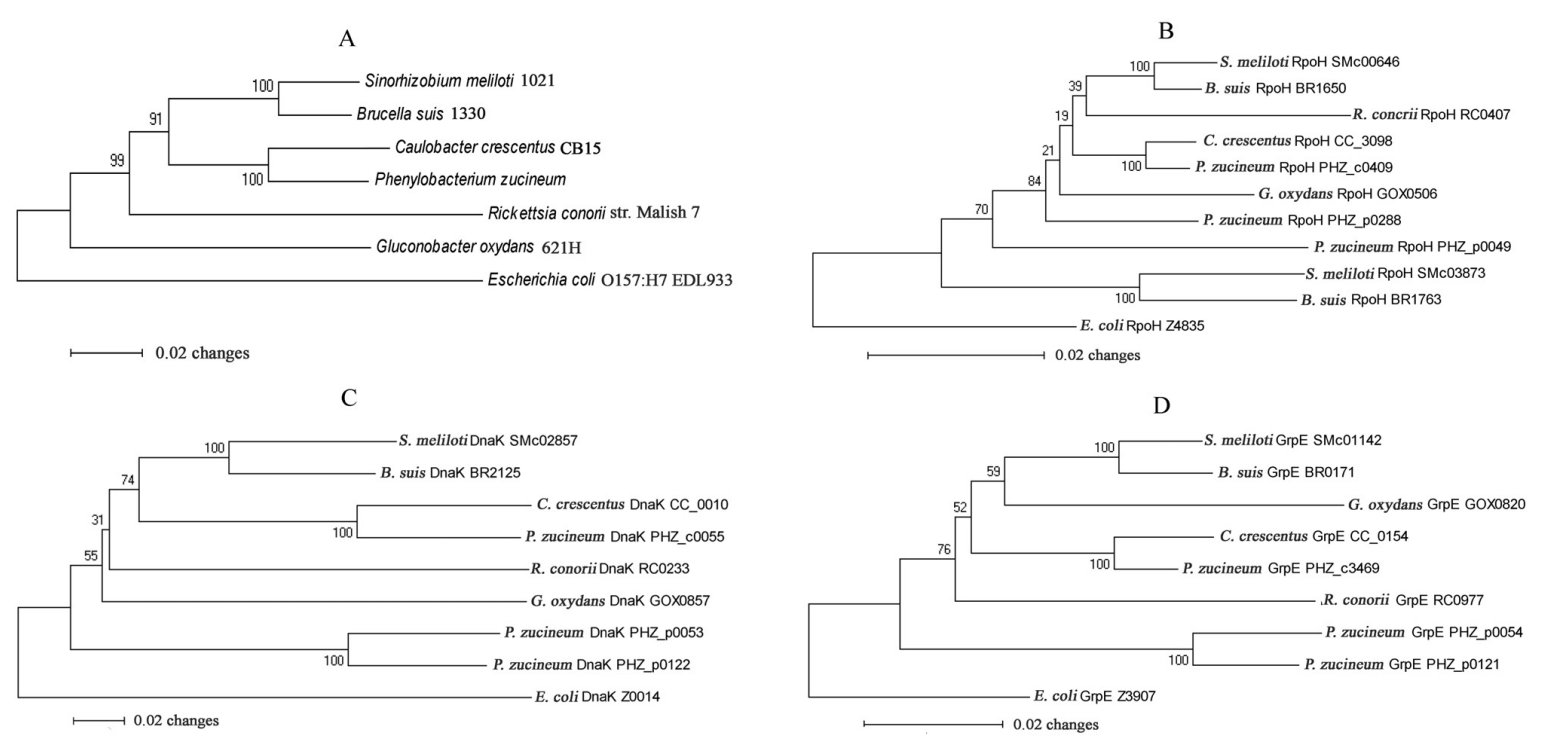
**Neighbor-joining trees of 5 representative alphaproteobacteria and *P. zucineum*, inferred from (A) 16S rRNA genes, (B) RpoH proteins, (C) DnaK proteins and (D) GrpE proteins**. The node labels are bootstrap values (100 replicates). The plasmid-encoded DnaK and GrpE of *P. zucineum *may have undergone a genus-specific gene duplication event (C &

To better understand the roles of proteins responsible for environmental transition, we computed the distributions of those proteins in 5 representative alphaproteobacteria with typical habitats (see methods – comparative genomics). Like other multiple bacteria and facultative bacteria, which can survive in multiple niches, *P. zucineum *encodes a higher fraction of ECFs, transcriptional regulators and two-component signal transduction proteins than obligate bacteria (Table 9). Notably, *P. zucineum *has the largest number of heat shock related proteins (Table 6), in comparison to the 5 representative alphaproteobacteria and 93 bacteria (data not shown). Among the plasmid-encoded heat shock related proteins are 2 RpoH (PHZ_p0049 and PHZ_p0288) and 2 DnaK-GrpE clusters (PHZ_p0053-0054 and PHZ_p0121-0122). Further phylogenetic analysis suggested that the plasmid-encoded DnaK-GrpE clusters may have undergone a genus-specific gene duplication event (Figure 3C &3D).

### Adaptation to an intracellular life cycle

To survive intracellularly, *P. zucineum *must succeed in adhering to and subsequently invading the host cell [12], defending against a hostile intracellular environment [13-16], and capturing iron at very low concentration [17].

It is well known that the pilus takes part in adhering to and invading a host cell [12]. We identified one pili biosynthesis gene (*pilA*) and 2 operons for pili biosynthesis (Table 11).

**Table 11:** Pilus proteins in the *P. zucineum *genome

Locus	5'-end	3'-end	Name	Gene symbol
PHZ_c0356	362,116	362,289	pilus subunit protein PilA	*pilA*
PHZ_c2992	3,412,800	3,413,318	Flp pilus assembly protein TadG	*tadG*
PHZ_c2995	3,415,220	3,415,468	Flp pilus assembly protein, pilin Flp	-
PHZ_c2996	3,415,532	3,416,023	Flp pilus assembly protein, protease CpaA	*cpaA*
PHZ_c2997	3,416,039	3,416,899	pilus assembly protein CpaB	*cpaB*
PHZ_c2998	3,416,899	3,418,350	pilus assembly protein CpaC	*cpaC*
PHZ_c2999	3,418,355	3,419,587	pilus assembly protein CpaE	*cpaE*
PHZ_c3000	3,419,594	3,420,991	pilus assembly protein CpaF	*cpaF*
PHZ_c3001	3,421,030	3,421,944	Flp pilus assembly protein TadB	*tadB*
PHZ_c3002	3,421,944	3,422,903	Flp pilus assembly protein TadC	*tadC*
PHZ_c3027	3,451,637	3,452,566	Flp pilus assembly protein CpaB	*cpaB*
PHZ_c3028	3,452,580	3,453,893	Flp pilus assembly protein, secretin CpaC	*cpaC*
PHZ_c3029	3,453,893	3,455,056	Flp pilus assembly protein, ATPase CpaE	*cpaE*
PHZ_c3030	3,455,059	3,456,489	Flp pilus assembly protein ATPase CpaF	*cpaF*
PHZ_c3031	3,456,489	3,457,445	Flp pilus assembly protein TadB	*tadB*
PHZ_c3032	3,457,492	3,458,391	Flp pilus assembly protein TadC	*tadC*

The genes involved in defense against oxidative stress include superoxide dismutase (PHZ_c0927, PHZ_c1092), catalase (PHZ_c2899), peroxiredoxin (PHZ_c1548), hydroperoxide reductase (*ahpF*, alkyl hydroperoxide reductase, subunit f, PHZ_c2725, *ahpC*, alkyl hydroperoxide reductase, subunit c, PHZ_c2724), and the glutathione redox cycle system (glutathione reductase [PHZ_c1740, PHZ_c1981], glutathione synthetase [PHZ_c3479], and γ-glutamylcysteine synthetase [PHZ_c0446, PHZ_c0523]).

Since intracellular free Fe is not sufficient to support the life of bacteria, to survive intracellularly, they must use protein-bound iron, such as heme and transferrin, via transporters and/or the siderophore system. The *P. zucineum *genome has one ABC type siderophore transporter system (PHZ_c1893-1895), one ABC type heme transporter system (PHZ_c0136, PHZ_c0139, PHZ_c0140), and 60 TonB-dependent receptors which may uptake the iron-siderophore complex (Table 12).

**Table 12:** TonB-dependent receptors in the *P. zucineum *genome

Annotation	Chromosome	Plasmid	COG category
TonB-dependent receptor	51	2	COG1629^1^
TonB-dependent receptor vitamin B12	3	0	COG4206^2^
TonB-dependent receptor	4	0	COG4771^3^

^1^COG1629, Outer membrane receptor proteins, mostly Fe transport

^2^COG4206, Outer membrane cobalamin receptor protein

^3^COG4774, Outer membrane receptor for monomeric catechols

### Comparative genomics between *P. zucineum *and *C. crescentus*

**Figure 4:**
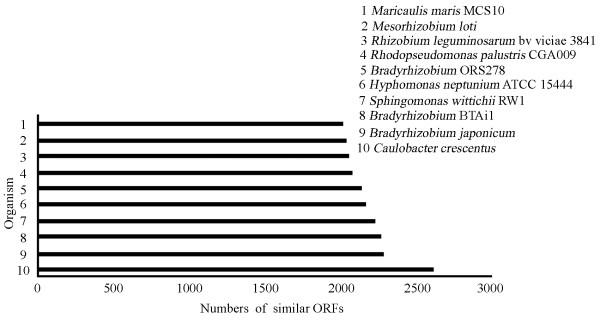
**List of top 10 complete sequenced bacteria closest to *P. zucineum***. All 10 are alphaproteobacteria. Among all the sequenced bacterial genomes, *C. crescentus *shares the greatest number of similar ORFs with *P. zucineum*

**Figure 5:**
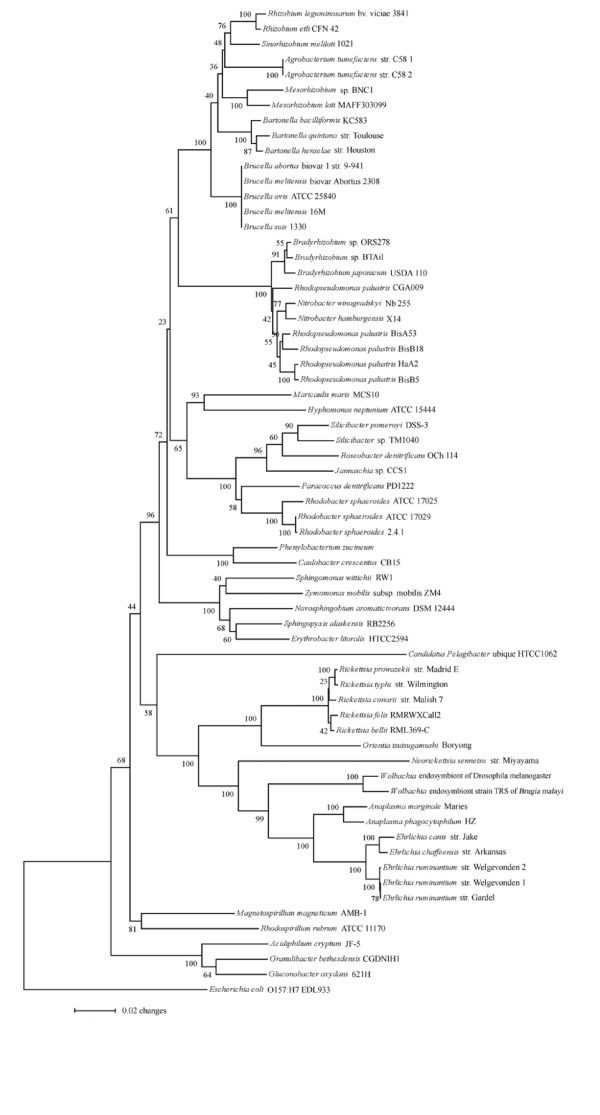
**Neighbor-joining tree of the alphaproteobacteria, inferred from 16S rRNA genes**. The node labels are bootstrap values (100 replicates). *C. crescentus *is phylogenetically the closest to *P. zucineum*.

Comparative genomic analysis demonstrated that *P. zucineum *is phylogenetically the closest to *C. crescentus *[18] (Figure 4), consistent with the phylogenetic analysis based on 16S RNA gene sequences (Figure 5).

Though the genome size and protein number of *P. zucineum *(4.37 Mb, 3,861 proteins) are similar to those of *C. crescentus *(4.01 Mb, 3,767 proteins), no large-scale synteny was found between the genomes. The largest synteny region is only about 30 kb that encodes 24 proteins. The conservation region with the largest number of proteins is the operon encoding 27 ribosomal proteins. In addition, the species share only 57.8% (2,231/3,861) of orthologous proteins. Categories J (translation, ribosomal structure and biogenesis), F (nucleotide transport and metabolism), and L (replication, recombination and repair) are the top 3 conservative COG categories between the species, sharing 88.01%, 81.67%, and 80.65% of the orthologs, respectively.

### Comparison of cell cycle genes between *P. zucineum *and *C. crescentus*

Since *P. zucineum *is phylogenetically closest to *C. crescentus*, and since the latter is a model organism for studies of the prokaryotic cell cycle [19,20], we compared the genes regulating the cell cycle between these species.

The cell cycle of *C. crescentus *is controlled to a large extent by the master regulator CtrA, which controls the transcription of 95 genes involved in the cycle [19,20]. On the other hand, *ctrA *is regulated at the levels of transcription, phosphorylation, and proteolytic degradation by its target genes, e.g., DNA methyltransferase (CcrM) regulates the transcription of *ctrA*, histidine kinases (CckA, PleC, DivJ, DivL) regulate its activity, and ClpXP degrades it. These regulatory 'loops' enable CtrA to precisely control the progression of the cell cycle.

*P. zucineum *has most of the orthologs mentioned above (Table 13). Among the 95 CtrA-regulated genes in *C. crescentus*, 75 have orthologs in the *P. zucineum *genome (Additional file 1). The fraction of CtrA-regulated genes with orthologs in *P. zucineum *(76.9%, 73/95) is significantly greater than the mean level of the whole genome (57.8%, 2,231/3,861), indicating that the CtrA regulatory system is highly conserved. Genes participating in regulating central events of the cell cycle, such as CcrM (CC0378), Clp protease (CC1963) and 14 regulatory proteins, except for one response regulator (CC3286), are present in the *P. zucineum *genome. The genes without counterparts in *P. zucineum *are mostly for functionally unknown proteins.

**Table 13:** Comparison of the signal transduction pathways regulating CtrA between the *P. zucineum *and the *C. crescentus*

	Locus	Length		Amino acid Identity (%)	Annotation
*C. crescentus*	*P. zucineum*	*C. crescentus*	*P. zucineum*		
CC0378	PHZ_c0577	355	359	80.00	modification methylase CcrM
CC1078	PHZ_c0933	691	663	67.22	cell cycle histidine kinase CckA
CC2482	PHZ_c2681	842	606	63.78	sensor histidine kinase PleC
CC1063	PHZ_c2712	597	504	53.83	sensor histidine kinase DivJ
CC3484	PHZ_c0218	769	769	67.66	tyrosine kinase DivL
CC2463	PHZ_c1309	130	121	89.26	polar differentiation response regulator DivK
CC1963	PHZ_c1817	202	205	80.19	ATP-dependent protease, ClpP subunit
CC1961	PHZ_c1814	420	420	90.47	ATP-dependent protease, ClpX subunit

**Figure 6:**
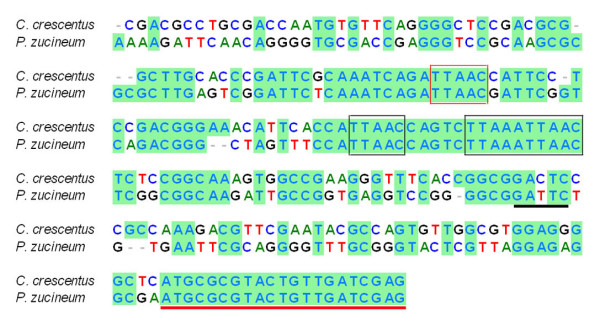
**Nucleotide acid sequence alignment of the *ctrA *promoter regions (-200 to +21) of *C. crescentus *and *P. zucineum***. Blue background: identical nucleotides; "-": gaps; red and black box: P1 and P2 promoter; black underline: motif recognized by CcrM; red underline: first 21 nucleotides starting with initial codon "ATG.".

Notably, the sequence of CtrA is strikingly similar between *P. zucineum *and *C. crescentus*, with 93.07% identity of amino acid sequence and 89.88% identity of nucleotide sequence. In addition, they share identical promoters (p1 and p2) [21] and the motif (GAnTC) recognized by DNA methyltransferase (CcrM) (Figure 6) [22], suggesting that they probably share a similar regulatory loop of CtrA.

Consistent with the results from *in silico *sequence analysis, the CtrA of *P. zucineum *can restore the growth of temperature-sensitive strain LC2195 (a CtrA mutant) of *C. crescentus *[23] at 37°C, indicating that the CtrA of *P. zucineum *can functionally compliment that of *C. crescentus *in our experimental conditions (data not shown).

Taken together, the comparative genomics of *P. zucineum *and *C. crescentus *suggests that the cell cycle of the former is likely to be regulated similarly to that of the latter.

### Presence of ESTs of the strain in human

Since *P. zucineum *strain HLK1^T ^can invade and persistently live in several human cell lines [1], we were curious about whether this microbe can infect humans. By blasting against the human EST database (dbEST release 041307 with 7,974,440 human ESTs) with the whole genome sequence of *P. zucineum*, we found 9 matched ESTs (Table 14), of which 3 were from a library constructed from tissue adjacent to a breast cancer, and 6 were from a library constructed from a cell line of lymphatic origin. The preliminary data suggest that *P. zucineum *may invade humans.

**Table 14:** Human ESTs matching the genome sequences of *P. zucineum*

Query GI	Sample origin	Query Length	Query Position		Chromosome Position		Score	E Value	Similarity (%)
						
			Begin	End	Begin	End			
14251638	Breast tissue^1^	226	41	175	1,276,914	1,277,048	204	2.00E-53	94.07
8261474	Breast tissue	116	1	108	1,277,042	1,276,937	167	2.00E-42	96.31
14251634	Breast tissue	142	19	134	1,277,054	1,276,937	204	1.00E-53	97.46
33194938	Lymphatic cell line^2^	441	8	441	1,029,575	1,029,142	749	0	96.77
33194696	Lymphatic cell line	652	8	652	1,029,575	1,028,931	1,166	0	97.67
33193754	Lymphatic cell line	654	8	654	1,029,575	1,028,929	1,191	0	98.15
7117824	Lymphatic cell line	405	7	405	1,558,831	1,558,433	735	0	98.25
33194587	Lymphatic cell line	638	7	638	2,864,470	2,863,838	1,191	0	98.89
7114909	Lymphatic cell line	347	6	347	3,498,624	3,498,283	654	0	99.12

^1^All of three sequences come from the library BN0075 containing 182 ESTs; the original dataset was produced by a modification of the EST sequencing strategy ORESTES (open reading frame expressed sequences tags)[44,45]

^2^All six sequences come from the library NIH_MGC_51 containing 2,381 ESTs; the original dataset was produced and released by the "Mammalian Gene Collection" project [46].

## Conclusion

This work presents the first complete bacterial genome in the genus *Phenylobacterium*. Genome analysis reveals the fundamental basis for this strain to invade and persistently survive in human cells. *P. zucineum *is phylogenetically closest to *C. crescentus *based on comparative genome analysis.

## Methods

### Bacterial growth and genomic library construction

*P. zucineum *strain HLK1^T^was grown in LB (Luria-Bertani) broth at 37°C and then harvested for the preparation of genomic DNA[1]. Genomic DNA was prepared using a bacterial genomic DNA purification kit (V-Gene Biotech., Hangzhou, China) according to the manufacturer's instructions. Sheared DNA samples were fractionated to construct three different genomic libraries, containing average insert sizes of 2.0–2.5 kb, 2.5–3.0 kb and 3.5–4.0 kb. The resulting pUC18-derived library plasmids were extracted using the alkaline lysis method and subjected to direct DNA sequencing with automated capillary DNA sequencers (ABI3730 or MegaBACE1000).

### Sequencing and finishing

The genome of *P. zucineum *was sequenced by means of the whole genome shotgun method with the phred/phrap/consed software packages [24-27]. Sequencing and subsequent gene identification was carried out as described in our earlier publications [28-30]. Briefly, during the shotgun sequence phase, clones were picked randomly from three shotgun libraries and then sequenced from both ends. 44,667 successful sequence reads (>100 bp at Phred value Q13), accounting for 5.47× sequence coverage of the genome, were assembled into 563 sequence contigs representing 60 scaffolds connected by end-pairing information.

The finishing phase involved iterative cycles of laboratory work and computational analysis. To reduce the numbers of scaffolds, reads were added into initial contig assembly by using failed universal primers as primers and by using plasmid clones that extended outwards from the scaffolds as sequence reaction templates. To resolve the low-quality regions, resequencing of the involved reads in low quality regions with universal primers and primer walking the plasmid clones were the first choice, otherwise, resequencing with alternate temperature conditions resolved the remaining low-quality regions. New sequence reads obtained from the above laboratory work were assembled into existing contigs, which yielded new contigs and new scaffolds connected by end-pairing information. Then, consed interface helped us to do nest round of laboratory work based on new arisen contig assembly. After about four iterative cycles of the above "finish" procedures to close gaps and to resolve the low-quality regions, the PCR product obtained by using total genomic DNA as template was sequenced from both ends to close the last physical gap. In addition, the overall sequence quality of the genome was further improved by using the following criteria: (1) two independent high-quality reads as minimal coverage, and (2) Phred quality value = Q40 for each given base. Collectively, 3,542 successful reads were incorporated into initial assembles during the finishing phase. The final assembly was composed of two circular "contigs", of which a smaller one with a protein cluster (including *repA*, *repB*, *parA *and *parB*) related to plasmid replication was assigned as the plasmid, and the larger one was the chromosome.

### Annotation

tRNA genes were predicted with tRNAscan-SE [31]. Repetitive sequences were detected by REPuter [32,33], coupled with intensive manual alignment. We identified and annotated the protein profiles of chromosome and plasmid with the same workstream. For the chromosome, the first set of potential CDSs in the chromosome was established with Glimmer 2.0 trained with a set of ORFs longer than 500 bp from its genomic sequence at default settings [34]. The resulting 5,029 predicted CDSs were BLAST searched against the NCBI non-redundant protein database to determine their homology [35]. 1,174 annotated proteins without the word "hypothetical" or "unknown" in their function description, and without frameshifts or in-frame stop codons, were selected as the second training set. The resulting second set of 4,018 predicted CDSs (assigned as "predicted CDSs") were searched against the NCBI non-redundant protein database. Predicted CDSs that accorded with the following BLAST search criteria were considered "true proteins": (1) 80% of the query sequence was aligned and (2) E-value ≤ 1e^-10^. Then, the ORFs extracted from the chromosome region among "true proteins" were searched against the NCBI non-redundant protein database. The ORFs satisfying the same criteria as true proteins were considered "true ORFs". Overlapping proteins were manually inspected and resolved, according to the principle we described previously [30]. The final version of the protein profile comprised three parts: true proteins, true ORFs, and predicted CDSs located in the rest of the genome. The translational start codon of each protein was identified by the widely used RBS script [36] and then refined by comparison with homologous proteins [30].

To further investigate the function of each protein, we used InterProScan to search against the InterPro protein family database [37]. The up-to-date KEGG pathway database was used for pathway analysis [38]. All proteins were searched against the COG database which included 66 completed genomes [39,40]. The final annotation was manually inspected by comprehensively integrating the results from searching against the databases of nr, COG, KEGG, and InterPro.

### Phylogenetic tree construction

16S rRNA genes were retrieved from 63 alphaproteobacteria, *P. zucineum *and *Escherichia coli *O157:H7 EDL933. A neighbor-joining tree with bootstrapping was built using MEGA [41]. The gammaproteobacterium *E. coli *was used as the outgroup to root the tree. To illustrate the evolutionary history of heat shock related proteins (RpoH, DnaK and GrpE), neighbor-joining trees based on the 16S rRNA genes and the above three proteins of 5 representative alphaproteobacteria (*Sinorhizobium meliloti *1021, *Brucella suis *1330, *C. crescentus *CB15, *Rickettsia conorii *str. Malish 7, *Gluconobacter oxydans *621H), *P. zucineum *and *E. coli *O157:H7 EDL933 were constructed.

### Comparative genomics

Sequence data for comparative analyses were obtained from the NCBI database . The database has 520 completely sequenced bacterial genomes (sequences downloaded on 2007/06/05). All *P. zucineum *ORFs were searched against the ORFs from all other bacterial genomes with BLASTP. The number of *P. zucineum *ORFs matched to each genome with significance (E value = 1e^-10^) was calculated.

To illustrate the contribution of transcriptional regulators and two-component signal transduction proteins to environmental adaptation, we compared the mean fraction of these two types of proteins in bacteria living in 6 different habitats, as described by Merav Parter [42]. These are: (1) obligate bacteria that are necessarily associated with a host, (2) specialized bacteria that live in specific environments, such as marine thermal vents, (3) aquatic bacteria that live in fresh or seawater, (4) facultative bacteria, free-living bacteria that are often associated with a host, (5) multiple bacteria that live in many different environments, and (6) terrestrial bacteria that live in the soil. For bacteria with more than one sequenced strain, we chose only one strain for the comparative study. The numbers of bacterial species in each group were: 26 obligate, 5 specialized, 4 aquatic, 28 facultative, 27 multiple, and 3 terrestrial. We annotated the proteins of these 93 species with the same workflow used for *P. zucineum *and calculated the mean fraction of transcriptional regulators and two-component signal transduction proteins.

In addition, we annotated the ORFs of 5 representative alphaproteobacteria with different habitats (multiple bacteria *S. meliloti *1021 and *G. oxydan*s 621H, facultative bacterium *B. suis *1330, aquatic bacterium *C. crescentus *CB15, and obligate bacterium *R. conorii *str. Malish 7) using the same workflow and computed the distributions of proteins involved in environmental adaptation.

### Ortholog identification

All proteins encoded by one genome were BLASTP searched against a database of proteins encoded by another genome [35], and *vice versa*. The threshold used in these comparisons was 1e^-10^. Orthology was identified if two proteins were each other's best BLASTP hit (best reciprocal match).

### Data accessibility

The sequences reported in this paper have been deposited in the GenBank database. The accession numbers for chromosome and plasmid are CP000747 and CP000748, respectively.

## Abbreviations

EST: Expressed Sequence Tag; KEGG: Kyoto Encyclopedia of Genes and Genomes.

## Authors' contributions

XH and SH designed the project; YL, XX, ZD, ZL, ZY and JS performed the research; SH and BZ contributed new reagents\analytical tools; YL, XX, and ZD analyzed the data; and XH, YL, and SH wrote the paper. All authors read and approved the final manuscript.

## Supplementary Material

Additional file 1 **Supplemental Table 1 **Comparison of genes directly regulated by CtrA between *P. zucineum *and *C. crescentus*. Click here for file
